# Automatic Detection of Grammatical Errors in English Verbs Based on RNN Algorithm: Auxiliary Objectives for Neural Error Detection Models

**DOI:** 10.1155/2021/6052873

**Published:** 2021-10-16

**Authors:** Yizhou He

**Affiliations:** School of Foreign Languages, Xinyu University, Xinyu, Jiangxi 338004, China

## Abstract

With the rapid development of neural network technology, we have widely used this technology in various fields. In the field of language translation, the research on automatic detection technology of English verb grammatical errors is in a hot stage. The traditional manual detection cannot be applied to the current environment. Therefore, this paper proposes an automatic detection technology of English verb grammatical errors based on recurrent neural network (RNN) algorithm to solve this problem. Firstly, the accuracy and feedback speed of traditional manual detection and recurrent neural network RNN algorithm are compared. Secondly, a detection model which can be calculated according to grammatical order combined with context is designed. Finally, when the output verb result is inconsistent with the original text, it can automatically mark the error detection effect. The experimental results show that the algorithm model studied in this paper can effectively improve the detection accuracy and feedback efficiency and is more applicable and effective than the traditional manual detection method.

## 1. Introduction

Internationally, with the rapid development of internationalization and globalization, English, as a global language, plays an important role in international trade, business cooperation, tourism, and other industries [1]. More and more people pay attention to the comprehensive development of English listening, speaking, reading, and writing. In recent years, with the remarkable development of information technology, the automatic system with the function of evaluation and feedback is widely welcomed by the second language learners, English learners, which can quickly, effectively, and accurately find the errors in English learning and learn English more efficiently and effectively [2, 3].

Computer-assisted language learning (CALL) system is one of the most effective language learning methods for automatic system. In the field of writing, with the significant development of deep learning methods and machine learning algorithms, English grammar error correction (GEC) systems are striding to identify and correct grammatical errors in a full range [4, 5], for example, the article, preposition, noun, verb, and other grammatical errors [6], such as the famous ESL assistant system designed and developed by Microsoft. From the perspective of theoretical research, generally speaking, the research methods used in GEC automatic detection include *n*-gram grammar, automatic classification, and machine translation model on the basis of grammar rule model. Researchers used these three different models for 26 types of grammatical errors in GEC system evaluation. For example, grammatical rules and *n*-gram grammar were used to identify verb tense and form errors, and grammatical rules and machine translation were used to identify verb missing errors. The results show that GEC system uses different models to automatically detect 26 kinds of grammatical errors, but the accuracy and recall rate of the final grammatical errors are low; for example, the recall rate of verb tense, verb form, and verb missing are 19.61%, 18.99%, and 15.19%, respectively [7]. Based on this, it is necessary to design a more optimized automatic detection method to improve the accuracy for different types of grammatical errors. As the core of English sentences, verbs are also the most complex part of speech in English learning. Therefore, this study focuses on the automatic detection technology of verb grammatical errors in English learning.

In addition, at present, in the aspect of spoken language, these systems can provide specific conversation and speech situations and are further developing to support free speech situations [8, 9]. Theoretically speaking, in the free speech situation, the English grammar error detection (GED) system mainly uses the automatic speech recognition (ASR) technology to complete the task due to the unknown discourse in advance [10, 11]. Among them, researchers have established a grammar error recognition system based on interactive CALL system language model optimization [12]. A CALL system for oral grammar practice based on ASR is proposed [13].

At the technical level, the original GEC and GED methods mainly rely on and use manual handwriting grammar rules and error grammar writing [14]. Later, with the development of computer science and the extensive use of deep learning, they focus on feature-based engineering, such as vocabulary grammar features [15], and use machine learning for modeling to facilitate automatic detection of English grammar errors. At the same time, the research on specific grammatical errors is more extensive. For example, modern research put forward the GED system for verb forms [16]. In the research of using machine learning to realize GED, in order to improve the reliability and accuracy, different neural networks and deep learning algorithms are gradually proposed [17, 18]. Some researchers first proposed a neural GED method based on bidirectional long-term and short-term memory neural network (BI LSTM) [19] and then extended and improved it [20]. According to the ASR technology used to realize GED, it is found that the results are mainly affected by two aspects: one is acoustic model (AM), and the other is language model (LM) [21]. Based on the former, the research on deep neural network has improved the speech recognition rate [20]. At the same time, the research based on the latter focusing on deep neural network has gradually emerged to improve the recognition rate of grammatical errors. Neural machine translation method was used to obtain training data of language modeling and identify grammatical errors, the mechanism of which is to use the method to generate sentences with grammatical errors from correct sentences [22]. Some people realized GED with the help of neural machine translation and applied statistical translation machine method and neural model [23]. On this basis, in order to propose a more reliable and accurate method, this study explores an automatic grammar error detection method based on recurrent neural network (RNN), and the research object is English verbs.

## 2. Automatic Detection of Grammatical Errors of English Verbs Based on RNN

### 2.1. Automatic Detection of Grammatical Errors in English Verbs Based on RNN

**Figure 1:**
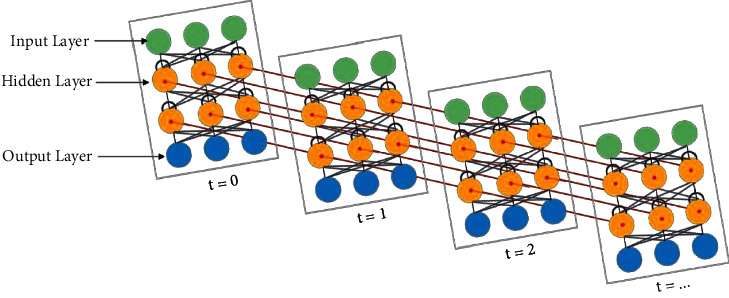
Recurrent neural network.

Different from the general neural network model, RNN is fully connected from the input layer to the hidden layer to the output layer [24]. The specific topology is shown in Figure 1.

As can be seen from Figure 1, a neural network with loops allows the RNN to store information. That is, RNN has memory for context information, can effectively infer information, and promotes the final result to be more accurate and reliable. Therefore, this paper applies it to the automatic detection of grammatical errors of English verbs, which is more helpful to achieve accurate recognition.

From the perspective of neurons, specifically the network includes an input layer *x*, a hidden layer *h*, and an output layer *o*. Correspondingly, the set of input units is {*x* (0), *x* (1),…, *x* (*t*)}. The implied unit set is {*h* (0), *h* (1),…, *h* (*t*)}. The output unit set is {*o* (0), *o* (1),…, *o* (*t*)}. At time *t*, the input vector *x* (*T*) corresponds to the hidden vector and the output vector formed by the neural network, which are *h* (*t*) and *o* (*t*), respectively.

In theory, the automatic detection model of grammatical errors of English verbs can be regarded as a kind of English language model LM.

Specifically, the training calculation process of RNN is as follows: in the English language training model, the input vector *x* (*t*) is formed by the concatenated vector *w* representing the current word time *t* and the output of the implicit layer of time *t* − 1. The specific expression is shown in the following formula:

(1) $$\begin{array}{c} x\left(t\right)=w\left(t\right)+h\left(t-1\right), \end{array}$$

where hidden vector *x* (*t*) is the memory of RNN and can obtain sequence information, which is the special difference between RNN and other general neural network algorithms. It is determined by the output of input vector *x* (*t*) and *t* − 1 hidden layer, so its calculation is cyclical. The specific calculation is shown in the following formula:

(2) $$\begin{array}{c} h\left(t\right)=f\left(U*x\left(t\right)+W*h\left(t-1\right)+\beta \right). \end{array}$$

**Figure 2:**
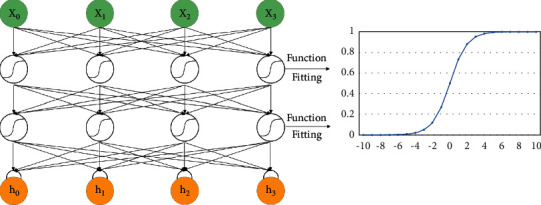
The sigmoid activation function fitting.

It is also worth noting that when *h* (−1) is used in the calculation, it is treated as a 0 vector, which is caused by the absence of *h* (−1). In addition, *f* in formula (2) is an activation function, which is generally a nonlinear function. The activation function sigmoid of exponential operation in deep learning is used for calculation. Its characteristic is that it can convert the real value into the output from 0 to 1. The example and image fitting are shown in Figure 2, which is shown in the following formula:

(3) $$\begin{array}{c} f\left(y\right)=\frac{1}{1+e^{-y}}. \end{array}$$

The output vector *o* (*T*) is only determined by the hidden vector of time *t*, that is, memory. The specific calculation is shown in the following formula:

(4) $$\begin{array}{c} o\left(t\right)=g\left(V*h\left(t-1\right)+\eta \right), \end{array}$$

where *V* is the network parameter and the total probability of the total time output is also the softmax activation function, which is used to make the data conform to the probability distribution to achieve the total probability of 1. Its calculation is expressed as in the following formula:

(5) $$\begin{array}{c} g\left(y\right)=\frac{e^{y}}{\sum_{k}e^{y_{k}}}. \end{array}$$

In addition, in the English language training model, including the verb grammar error automatic detection model, the RNN-based experimental setup is very important. For RNN, initialization is not the most critical when it is used to process data of large order of magnitude. Therefore, the implicit vector *s* (0) is set to a vector composed of smaller values similar to 0.1. The input vector, such as formula (1), is determined by the 1-of-*N* code and the previous hidden layer, so the size of the vector is determined by the size of the vocabulary plus the size of the hidden layer. The scale of hidden layer is usually 30–500 hidden units. Specifically, the scale of reference experimental training data is determined; that is, the larger the scale of training data is, the larger the scale of hidden layer is.

### 2.2. Design of Automatic Detection of Grammatical Errors of English Verbs Based on RNN

In general, the automatic detection of English grammar based on neural network algorithm is comprehensive, which is intended to cover all types of grammatical errors. However, experimental research shows that its reliability and validity are low, which greatly hinders the development of related programs and applications. Verbs are the core of English sentences. It is very important for second language learners to find grammatical errors of English verbs comprehensively and effectively. In this study, through the study of learners' written and oral materials, it is found that verb grammatical errors are often manifested in verb tense, verb absence, and verb form. Therefore, the design of automatic syntax error detection focuses on these three types.

For these three kinds of grammatical errors, the model based on RNN needs to machine-learn the embedding function of verb context, accurately predict the verb form according to the context, and form the target word. If the predicted words are different from the original form, the verbs in the original sentence are marked as errors. At the same time, the model based on RNN can realize the training form from the beginning word to the target verb, from the ending word to the target verb, and even from the context to the target word in a fixed scale.

**Figure 3:**
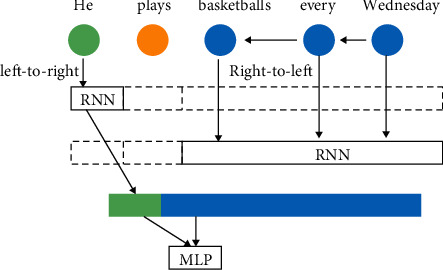
The language model based on RNN for verb grammar detection.

Figure 3 is taken as an example. First of all, Figure 3 involves two RNN models: “plays” in English sentences is the target word. One RNN model trains the initial word “he” from left to right, and the other RNN model trains the ending word “Wednesday” from right to left.

Furthermore, the training based on RNN model is expressed by mathematical language as shown in the following formula:

(6) $$\begin{array}{c} bi\text{RNN}\left(w_{1:n},i\right)=l\text{RNN}\left(w_{1:i-1}\right)⊕r\text{RNN}\left(w_{i+1:n}\right). \end{array}$$

Formula (6) represents the definition form of the target verb *w*_*i*_ for an English sentence *w*_1:*n*_. *l*RNN represents the RNN model read from left to right in a given English sentence. Similarly, *l*RNN represents the RNN model read from right to left. l or *r* represents two different embedding methods, respectively.

When the RNN-based input layer, hidden layer, and output layer are processed, the output of the final result still needs to enter the last layer of neural network design, full connection layer. In this paper, multilayer perceptron (MLP) is applied, including input layer, hidden layer, and output layer. That is, all the neurons in the former layer are connected with those in the latter layer.

For MLP, its forward propagation needs activation function to prevent multilayer network from degenerating into single-layer network. At the same time, it also needs activation function in the hidden layer to reduce network degradation, so that the neural network can approach the nonlinear function. Therefore, when the two RNNs are finished, MLP is used to predict the target verb or the form of target verb, such as present participle or past participle. We need to use the following formula:

(7) $$\begin{array}{c} \text{MLP}\left(x\right)=\text{soft}\max\left(\text{Relu}\left(L\left(x\right)\right)\right). \end{array}$$

**Figure 4:**
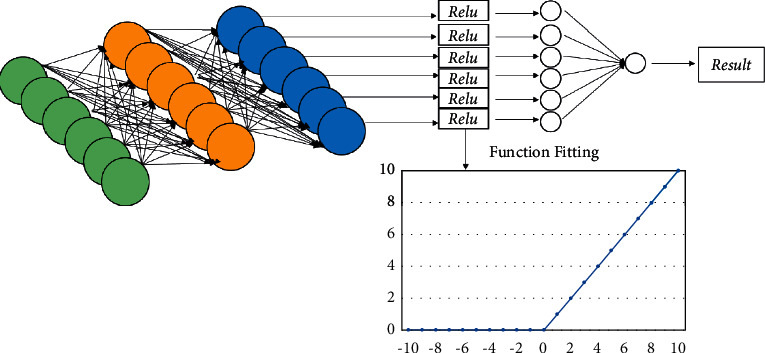
The relu activation function fitting.

The form of softmax activation function in formula (7) is consistent with that in formula (5). Relu is the activation function of revised linear unit (relu). Its function and fitting are shown in Figure 4. The function is expressed as in the following formula:

(8) $$\begin{array}{c} \text{Relu}\left(x\right)=\max\left(0,x\right). \end{array}$$

In formula (7), the original calculation of hidden layer adopts the form of full connection linear operation, which is expressed as in the following formula:

(9) $$\begin{array}{c} L\left(x\right)=Wx+b. \end{array}$$

According to the above, the processing of RNN-based input layer, hidden layer, and output layer and MLP full connection neural network, combined with formulas (6) and (7), the calculation of hidden layer can be divided into two parts. The final result of each output layer of recurrent neural network is expressed as in the following formula:

(10) $$\begin{array}{c} Y=\text{MLP}\left(bi\text{RNN}\left(w_{1:n},i\right)\right). \end{array}$$

In formula (10), *y* is the target verb or the form of target verb predicted by each output layer of recurrent neural network. Therefore, each time, the error vector can be calculated according to the cross entropy principle, and the output layer weight can be updated according to the standard BP neural network algorithm, which is expressed as in the following formula:

(11) $$\begin{array}{c} \text{error}=Z-Y. \end{array}$$

In formula (11), *Z* represents the target verb or target verb form that should be predicted in English sentences, and *Y* is the meaning represented in formula (10). Based on the RNN neural network algorithm and MLP hidden layer of multilevel memory processing, when the neural network output of the prediction of the target verb or the target verb form is not completely consistent, this inconsistency will be marked as grammatical errors, so as to realize the automatic detection of English verb grammatical errors based on RNN algorithm. In particular, for the three different grammatical errors of verb tense, verb absence, and verb form, the classification label is marked as ˆ *y*. The objective equation of training is expressed as in the following formula:

(12) loss=1n∑i=1n^yilogyi,

where *n* is the number of training samples. In addition, specifically, the definition of *Y* in formula (10) is different, which is comprehensively listed in Table 1.

**Figure 5:**
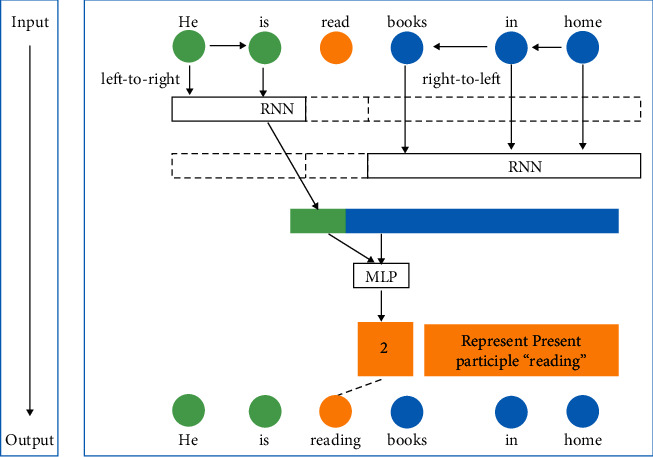
The verb grammar error detection based on RNN language model.

According to Table 1, in the verb form grammar error detection model based on RNN algorithm, the value of *Y* represents different verb forms; for example, 0 represents basic form, 1 represents past word segmentation, and 2 represents word segmentation. Taking the English sentence “he is read books in home” as an example, the English verb form grammar error model based on RNN algorithm is used for automatic detection. The input and output results are shown in Figure 5.

When the output in Figure 5 is inconsistent with the verb form “read” given by the original English sentence, the model will automatically judge the grammatical error of the original verb form “read” according to the design.

## 3. Experimental Evaluation of Automatic Detection of Grammatical Errors of English Verbs Based on RNN

### 3.1. Experimental Design of Automatic Detection of Grammatical Errors of English Verbs Based on RNN

In the English language model, the learner corpus with manually labelled grammatical errors is generally used as the standard to compare the automatic detection of grammatical errors by machine learning with manually labelled grammatical errors to evaluate the effectiveness of the design or technology of automatic detection of grammatical errors by machine learning. In the whole process of comparison, it is necessary to analyze the accuracy of the two in the process of verb grammatical error detection. The accuracy calculation is shown in the following formula:

(13) $$\begin{array}{c} \text{Precision}=\frac{\sum_{i=1}^{n}\left|e_{i}\cap g_{i}\right|}{\sum_{i=1}^{n}\left|e_{i}\right|}. \end{array}$$

After obtaining the accuracy of the two methods of detection, it is also necessary to compare the feedback rate of the automatic detection system. The calculation formula is as follows:

(14) $$\begin{array}{c} \text{Recall}=\frac{\sum_{i=1}^{n}\left|e_{i}\cap g_{i}\right|}{\sum_{i=1}^{n}\left|g_{i}\right|}. \end{array}$$

In (13) and (14), *e*_*i*_ is the set of machine learning automatic detection outputs of grammatical errors and *g*_*i*_ is the set of manual markings of grammatical errors. Meanwhile, the intersection of sets *e*_*i*_ and *g*_*i*_ in the formula is defined as in the following formula:

(15) $$\begin{array}{c} e_{i}\cap g_{i}=\left\{e\in e_{i}|\exists g\in g_{i},\text{match}\left(g,e\right)\right\}. \end{array}$$

Furthermore, taking the English sentence “he play basketballs and swam every weekend” as an example, the accuracy of automatic detection of grammatical errors is 100% and the recall rate is 50% under the assumption that the verb grammatical error set *g* = {play⟶plays, swam ⟶swims} is manually labelled and the final verb grammatical error set *e* = {play⟶ plays} is detected by machine learning. Theoretically, the higher the value of these two indicators, the higher the effectiveness of automatic detection of verb grammatical errors by machine learning. However, according to formulas (13) and (14), there is a contradiction between accuracy and recall. When the recall rate is higher, that is, more grammatical errors and incomplete errors of verbs are detected and marked automatically, the accuracy will be reduced.

Therefore, in this paper, in order to solve this problem and highlight the fact that it is more tendentious to accurately identify errors than to try to cover more errors in practical application, *F*_0.5_ is used as an index to assign a higher weight to the accuracy rate, and this index is used as the main measure of model training in this paper. The calculation of *F*_0.5_ is expressed as in the following formula:

(16) $$\begin{array}{c} F_{0.5}=\frac{\left(1+0.5^{2}\right)*\text{Recall}*\text{Precision}}{\text{Recall}+0.5^{2}*\text{Precison}}. \end{array}$$

As for the data set of the training experiment, this paper uses the classic conll-2014 data set. The English grammatical errors covered in this training data set include articles, determiners, prepositions, noun forms, verb forms, subject predicate consistency, pronouns, sentence structure, punctuation, capitalization, and other types. It should be pointed out that the automatic detection of English grammatical errors based on RNN algorithm is designed for three types of grammatical errors of verbs in English sentences. Therefore, in the classic conll-2014 data set, this paper selects English sentences with grammatical errors of verb form as the standard data set for training and testing, The training set and the test set are randomly selected according to the ratio of 8 : 2. At the same time, it should be emphasized that, before the evaluation experiment, in order to further improve the performance of the results, the verb words whose occurrence probability is less than the set threshold in the data set are integrated into a special rare marker set *α*. Specifically, the occurrence probability is calculated as in the following formula:

(17) $$\begin{array}{c} P\left(w\left(t+1\right)|w\left(t\right),h\left(t-1\right)\right)=\left\{\begin{array}{cc} \frac{Y_{\partial }\left(t\right)}{C_{\partial }}, & w\left(t+1\right)\in \partial ,\\ Y\left(t\right), & \text{Otherwise.} \end{array}\right. \end{array}$$

In formula (17), the value of *C*_*α*_ is the number of words whose occurrence times are lower than the set threshold, so by formula (17), the probability is evenly distributed among all rare verb words. In addition, in the experiment, the initial word input is embedded by word set embedding and kept updated during the training process. At the same time, the rule size is set to 300, while the hidden layer size needs to be set by using the training data set experiment to select a more appropriate value. At the same time, Stanford corenlp is used to locate the target words and verbs in English sentences. When the predicted result is different from the original English sentence, or the probability is greater than the present development value, the verb grammatical error will be recognized. In addition to the above experimental design and principles, this paper emphasizes the notion that the experiment focuses on the grammatical errors of verb forms. According to the listing and setting in Table 1, the verb forms are positioned as basic forms, past participles, and present participles. According to the knowledge of English grammar, locating grammatical errors in the form of verbs usually needs the help of English sentence context which is relatively far away from the target verb; for example, in “to prevent other accidents from happy…,” “prevent from” usually appears as a fixed phrase, and locating the target verb “prevent” needs the help of “from” in the context below. Therefore, in order to identify grammatical errors of English verb forms more accurately, the whole sentence is used as the context.

### 3.2. Experimental Evaluation and Analysis of Automatic Detection of Grammatical Errors of English Verbs Based on RNN

**Figure 6:**
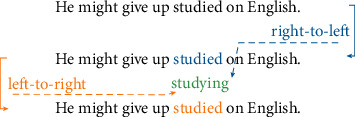
The output of verb form error detection based on RNN language model.

In the automatic detection of grammatical errors of English verbs, the grammatical errors of verb forms shown in Table 2 and Figure 6 as No. 2 example should be recognized, and the grammatical errors of basic verb forms, past participles, and present participles can be identified respectively.

**Figure 7:**
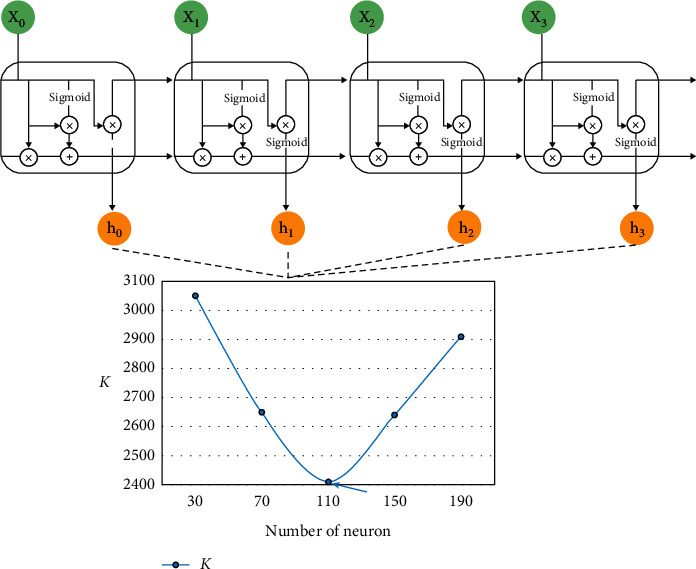
The comparison of *K*-value in different numbers of neuron.

In addition, in the design of the previous chapter, it is mentioned that the hidden layer of RNN plays an important role in the whole neural network, and the number of hidden neurons with memory effect will also affect the evaluation results in the experiment. Therefore, in order to further explore the most suitable number of hidden layer neural units in this experiment, we use the result *K* obtained from exponential operation of training objective equation as the evaluation index to set up a comparative experiment, which is calculated as in formula (18), and the final result is shown in the example in Figure 7.

(18) $$\begin{array}{c} K=\exp\left(\text{loss}\right). \end{array}$$

**Figure 8:**
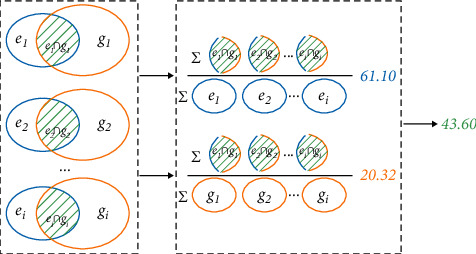
The expression of 3 different evaluation indexes.

Theoretically, the smaller the *K* value is, the better the training effect of automatic recognition of English language model is, and vice versa. It can be seen from the observation of Figure 7 that when the number of hidden layer neurons in RNN increases from 30 to 110, the value of *K* shows a decreasing trend, and the evaluation effect is getting better and better, which reflects that the performance of English model based on RNN algorithm for automatic detection of grammatical errors of English verb forms is gradually improved. When the number increases from 110 to 190, the value of *K* increases, and the evaluation effect becomes worse. Based on this, we think that when the number of neurons in the hidden layer is 110, the experimental effect is the best, so the following experiment is carried out with the number of neurons of 110, and the results are explained. On the whole, based on the algorithm model and experimental design, the results of evaluating grammatical errors of English verb form from Precision, Recall, and *F*_0.5_ are listed in Table 3. The performance of 3 indexes is shown in Figure 8.

**Figure 9:**
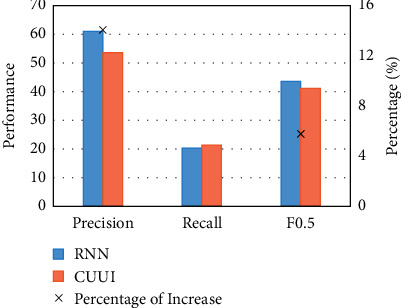
The comparison of results between RNN and GUUI.

According to the absolute value of evaluation index in Table 3, the design of automatic detection of grammatical errors of English verbs based on RNN algorithm designed in this study has a limited understanding of the evaluation performance of grammatical errors in verb forms in classic conll-2014 data set. Therefore, the evaluation results of conll-2014 verb forms using CUUI as the best classifier method are compared with the evaluation results in this paper. The details are shown in Figure 9.

From the comprehensive view of Figure 9, the absolute value level index increased by 2.38, the accuracy rate index increased by 7.50, the recall rate index slightly decreased, and the relative value level index and accuracy rate increased by 5.77% and 13.99%, respectively. In addition, it can be found that when the accuracy rate increases, it is often accompanied by the decline of recall rate. Further experiments show that, when exploring the language model of English grammar recognition based on neural network algorithm, we need to comprehensively consider the contradiction between the accuracy rate and recall rate and evaluate the experimental results. At the same time, it also further explains the significance of the research on the comprehensive evaluation index and evaluation index.

**Figure 10:**
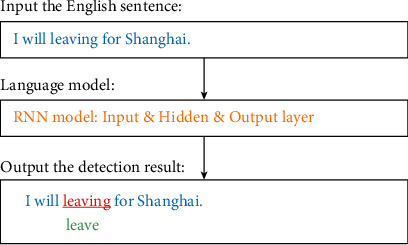
The detection result based on the test data sets.

When detecting grammatical errors of English verbs, taking completion grammatical errors as an example, automatic detection and error correction can be realized, as shown in Figure 10. Therefore, the automatic verb syntax error detection technology based on RNN algorithm proposed in this paper has significantly improved the accuracy and applicability compared with the traditional neural network algorithm and manual marker detection. Finally, it shows the result trend similar to the training set. According to the evaluation of the experimental training results, the algorithm model designed in this paper is effective in the automatic recognition of English sentence and verb grammatical errors and can be further applied to practice.

## 4. Conclusion

In recent years, with the rise of neural network research and the excellent development of information technology, English grammar error automatic recognition system with the function of evaluation and feedback is widely welcomed by English second language learners. Among them, efficient and accurate automatic detection technology is the key and difficult point. In addition, verbs are the soul of English sentences, so it is very important to accurately identify English verb grammar errors, Therefore, this paper studies and designs the automatic detection technology of English verb grammatical errors based on RNN algorithm. This language model involves two sub-RNN algorithm language models, including input layer, hidden layer, and output layer, from the beginning of English sentence to the target verb and from the end to the target verb. The results processed by RNN will finally enter the last layer NLP of neural network and output the final predicted verb or verb form. Combined with the context of English sentence, the correct verb or verb form is predicted and compared with the original sentence result to realize automatic error detection. According to the design, this language model can identify three types of verb grammatical errors: verb tense, verb missing, and verb form. The experimental results using the classic conll-2014 data set as the training set and test set show that the accuracy, recall rate, and *F*_0.5_ evaluation index of the design based on RNN algorithm are 61.10%, 20.32, and 43.60, respectively. Compared with the evaluation results of CUUI method, the accuracy and *F*_0.5_ are improved by 13.99% and 5.77%, respectively. It is proved that the algorithm model designed in this paper is effective in the automatic recognition of grammatical errors of English sentences and verbs and can be further applied in practice. The followup research will introduce attention mechanism into the language model to improve the expressiveness of the model.

## Figures and Tables

**Table 1:** Verb formal syntax error detection based on RNN algorithm.

Errors	Values of *Y*	Meanings
Tense	0	Present tense
Tense	1	Past tense
Tense	2	Future tense
Tense	3	Perfect tense
Missing	0	Normal
Missing	1	Absence
Form	0	Basic form
Form	1	Past participle
Form	2	Present participle

**Table 2:** Examples of verb form errors.

No.	Input	Output
1	He often plays footballs every Sunday.	Plays
2	He might give up studied on English.	Studying
3	I will leaving for Shanghai.	Leave

**Table 3:** Performance of the results.

Precision	Recall	*F*_0.5_
52.2	16.78	35.20
61.10	20.32	43.60
73.4	24.23	49.76

## Data Availability

The data used to support the findings of this study are available from the corresponding author upon request.
